# Grit but Not Help-Seeking Was Associated with Food Insecurity among Low Income, At-Risk Rural Veterans

**DOI:** 10.3390/ijerph20032500

**Published:** 2023-01-31

**Authors:** Yue Qin, Douglas A. Sneddon, Shelley MacDermid Wadsworth, Dave Topp, Rena A. Sterrett, Jake R. Newton, Heather A. Eicher-Miller

**Affiliations:** 1 Department of Nutrition Science, Purdue University, West Lafayette, IN 47907, USA; 2 Department of Human Development and Family Studies, Purdue University, West Lafayette, IN 47907, USA

**Keywords:** food security, rural community, veterans, grit, help-seeking, resource use, psychological factors, nutrition policy, pilot project

## Abstract

Rural veterans have poorer health, use healthcare services less often than their urban counterparts, and have more prevalent food insecurity than average U.S. households. Food insecurity and resource use may be influenced by modifiable psychological attributes such as grit and help-seeking behaviors, which may be improved through interventions. Grit and help-seeking have not been previously evaluated among rural veterans. Thus, this cross-sectional study evaluated the hypothesis that grit and help-seeking were associated with food insecurity and the use of resources. Food security, resource use, grit, and help-seeking behavior were assessed among rural veterans (≥18 years) from five food pantries in southern Illinois counties (*n* = 177) from March 2021 to November 2021. Adjusted multiple regression was used to estimate the relationship between the odds of food insecurity and the use of resources with grit and help-seeking scores. Higher grit scores were significantly associated with lower odds of food insecurity (OR = 0.5, *p* = 0.009). No other associations were detected. The results provided evidence to inform the content of future educational interventions to improve food insecurity and address health disparities among rural veterans by addressing grit. The enhancement of psychological traits such as grit is related to food security and has the potential to benefit other aspects of well-being.

## 1. Introduction

Over 4.7 million veterans live in rural areas of the U.S. [1]. These veterans are in poorer health [2,3], disproportionately likely to have service-connected disability ratings above 50% [4], and have more severe mental health disorders [5] compared to their suburban and urban counterparts. Despite poorer health conditions, rural veterans are less likely to use healthcare services, which is likely due to less access to resources from transportation barriers [4]. Compared with 10.5% average household food security among U.S. adults [6] and 12.1% in rural areas [6], up to a quarter of U.S. veterans [7,8], representing 4.5 million people [9], were estimated to have inadequate access to enough food, known as food insecurity [10,11]. Living in this situation has been associated with poor nutrient intake and increased risk for various chronic diseases such as diabetes, hypertension, and mental health disorders [12]. Government assistance programs, such as the Supplemental Nutrition Assistance Program (SNAP) and Temporary Assistance for Needy Families (TANF), are designed to help those among the lower-income population by providing benefits for meeting basic needs [13]. Unfortunately, rural veterans specifically and veterans overall face many barriers to using these resources, such as limited access to transportation, a high likelihood of disability, few years of education, low rates of employment, the stigma associated with seeking help, and concerns related to the reporting and recording of health problems [14,15]. As a result, this at-risk population is less likely than non-veteran populations to utilize the assistance programs and healthcare resources that could help them improve their health conditions [16,17]. The high burden of disease faced by rural veterans highlights the importance of assessing and improving help-seeking behavior, which is linked to motivation and performance [18] as well as grit, a learned behavior that is advantageous for facing adversities [19]. Rates of help-seeking are low, especially among individuals suffering from psychiatric disorders [20], and low help-seeking is found in veteran populations who tend to underuse mental health services and are underserved in general [21,22,23]. Other psychological attributes, such as determination and perseverance for long-term goals, or “grit”, may be linked to maintaining food security [24,25]. Similar to resiliency, grit also captures prospective future planning [26]. Furthermore, grit may be improved through interventions [19,27,28] to support preserving food security, yet only two studies have examined and found inverse associations between reported grit and food insecurity [25,29] and none have evaluated the link of help-seeking behavior with food insecurity.

Interventions targeting grit among individuals at risk for high stress and lacking protective resources tend to be effective over time [28]. Similarly, interventions focusing on mental health and wellness promotion have increased help-seeking behaviors and intentions to seek help among adolescents [30] and adult college students [31]. To our knowledge, no intervention program targeting food security improvement has focused on grit and help-seeking behavior, yet their inclusion and fostering of these qualities may be a key link to health in veteran populations. The objectives of the current project were to examine the association of grit and help-seeking behavior with food insecurity and the use of resources among rural veterans from five food pantries in southern Illinois counties using a cross-sectional sample. The authors hypothesized that grit and help-seeking would each be linked to food insecurity and the use of resources.

## 2. Materials and Methods

### 2.1. Design and Participants

Rural veterans ≥18 years were recruited from March 2021 to November 2021 at five food pantries located in southern Illinois counties with high rurality scores (rurality scores ≥ 5 [32]) with large populations of low-income veterans [33]. Baseline data and a cross-sectional design were used but the data were drawn from a larger, longitudinal community food pantry pilot intervention called Reaching Rural Veterans (RRV) to improve food security and resource use, of which a version was previously carried out in Indiana and Kentucky [34]. Specifically, food pantries were selected from target counties based on several factors—rurality, need and sustainability, plans for sustained community partnerships, and budget to extend services to veterans, as described previously—and invited to participate in the project [34]. Next, recruited food pantry staff and volunteers received training and education to improve their awareness and knowledge of veterans’ needs and how to attract veterans to the pantry. The recruited pantries then implemented these marketing strategies to attract veterans to their food pantries at special veteran-targeted outreach events where veteran-relevant service organizations were represented. The outreach events were similar across the recruited pantry sites. At these events, the service organizations were represented by booths that veterans and their families could visit to learn about resources and their potential eligibility. Food pantry services were also offered at these events. Upon departure from these outreach events, food pantry patrons who self-identified as veterans were asked to fill out a questionnaire that included the measures discussed in more detail in the following section. The project team members screened the collected responses on site to ensure individuals met the inclusion criteria (≥18 years, served in the active or reserve U.S. military, can speak and read English, visit food pantries during the project period, and agreed to participate) to be included in the sample for analysis. The project was reviewed and considered exempt from Human Subjects Research by Purdue University’s Institutional Review Board.

### 2.2. Measures

Veteran participants were invited to self-complete or receive interviewer assistance from RRV staff, to complete questionnaires that queried demographic information and could be used to classify food security, assistance program and resource use, grit, and help-seeking behavior.

Missing responses were present among two of the independent variable assessments, grit and help-seeking, and for one of the outcome variable assessments, food security. Even though the project team adapted novel strategies to recruit and engage veterans, recruitment during the COVID-19 pandemic restrictions made it challenging to ensure adherence to protocols such as completing the surveys. For example, a portion of the surveys were taken home by the veterans and mailed back after completion with missing responses. Furthermore, onsite project staff received complaints about the length of the survey, which may have led to skipping questions among those who completed the surveys at the pantry. In order to utilize the available data collected from the current pilot project and retain statistical power, while making the best judgments to draw accurate conclusions, the imputation of certain missing data was completed to yield the most conservative outputs. Details of imputation are described below for each measure.

Grit was assessed using the (8-item) Short Grit Scale, which has been previously validated in adults [24]. Specifically, the participants were presented with 8 statements to describe themselves, such as “setbacks don’t discourage me” and “new ideas and projects sometimes distract me from previous ones”, and were asked to select an answer from the 1 to 5 response choices ranging from “very much like me” to “not like me at all” [24]. Scores of 1–5 were assigned to each response (4 of the 8 items were reverse scored), and the responses were averaged to generate the final score, which ranged from 1 to 5 [24]. Higher scores indicated more grit [24]. Participants with missing responses on the Grit Scale were few (*n* = 17). Consistent with other variables, grit mean scores were calculated for participants who responded to at least 75% of the items (6 items) on the Grit Scale [35]. Given that our data were not missing at random, available item analysis [36] was the most appropriate tool. Mean scale scores could not be computed for 7 participants.

Help-seeking behavior was measured using the modified General Help-Seeking Questionnaire (GHSQ), a validated and widely utilized questionnaire in psychological research to capture help-seeking intentions [37]. The GHSQ used a standard format of “If you were having [type of problem], how likely is it that you would seek help from the following people?”, where the type of problem could be modified to fit the project purpose [37]. We assessed 10 potential sources of help, such as “intimate partner”, “friend”, or “parent”, and for each, participants were asked to select from a 7-point scale ranging from “extremely unlikely” to “extremely likely”. The questionnaire administered in the current project included “having a personal or emotional problem” to assess the general help-seeking behaviors of these rural veterans. Participants with missing responses numbered 80. Scale averages were calculated for all participants who responded to at least 75% of the items (8 items); a higher score indicated more help-seeking behaviors. Scale means could not be calculated for 13 participants.

Food security was measured using the USDA 6-item Household Food Security Survey Module (HFSSM) [38]. Affirmative answers (“yes”) were summed to generate raw scores, with a raw score of 0–1 categorized as “food secure” and 2–6 as “food insecure” [38]. The category “food insecure” was further broken down into low food security (raw score of 2–4), where the quality of food is less than in food-secure situations, and very low food security (raw score of 5–6), where the quality and quantity of food is less than in food-secure situations [38]. Most participants had missing items within the 6-item HFSSM (*n* = 107). Imputation was completed only on those with 1 missing response, following the USDA guidance for imputation for the 18-item scale [38] to yield a conservative classification. The questions were reordered based on severity. The missing responses were coded as “yes” or “no” based on the responses before and after the missing values [38]. When missing values were found after the last non-missing response, they were not allowed to be counted as “yes”, and thus were not added to the raw score to calculate the food security level, potentially influencing the results towards a less severe range of food security.

Resource use was determined by type (yes or no for each resource) and tallied (total *n* of resources used) and included: Veterans Affairs healthcare (missing *n* = 36), disability payments (missing *n* = 21), Veteran Affairs disability benefits (missing *n* = 83), veteran pension (missing *n* = 11), employer pension/retirement fund (missing *n* = 13), social security benefits (missing *n* = 6), TANF (missing *n* = 15), employment compensation (missing *n* = 15), general assistance from the township trustee (missing *n* = 15), SNAP (missing *n* = 10), free meal/soup kitchens (missing *n* = 15), and free/reduced-price meals at school/childcare (missing *n* = 15). To be conservative, missing responses were coded as “No” when calculating the sum of program use.

### 2.3. Statistical Analysis

Characteristics (such as age, race, income, etc.) of the sample were described in numbers and percentages, and their relationships to each outcome variable, food security and the use of resources, were assessed using analysis of variance tests. Logistic and Poisson regression models were used to determine the relationship between food insecurity and the use of resources, respectively, with grit and help-seeking in the respective models. None of the characteristic variables were significantly related to either of the outcomes of interest. Multiple linear and logistic regression models created for the main results were adjusted for covariates age and sex, based on previous literature [39,40,41,42]. Model assumptions were evaluated and supported. The significance level was set as α < 0.05.

## 3. Results

The socio-demographic characteristics and resource use of the sample are shown in Table 1. A total of 235 veterans visited the food pantries during the duration of the program and were invited to participate in the sample. The veterans who chose to complete the assessments and were included in the socio-demographic analysis numbered 177. After imputation, *n* = 162 for food security, *n* = 170 for grit, and *n* = 164 for help-seeking were included in each respective analysis. A high proportion of the sample was male (89%), white (92%), over 65 years (65%), with education below college (91%), unemployed or out of the labor force (85.2%), married or living with a partner (57%), without adult children (84%), with fewer than three adults in the household (89%), and with a 12-month household income of less than $30,000 (60%). The participants predominantly served in the Army (67%), did not serve in the Guard/Reserve (70%), and served for an average of 5.6 years.

The resource use of the sample is presented in Table 2. The majority of the sample used several veteran-related resources, such as Veterans Affairs healthcare (77%) and Veterans Affairs disability benefits (60%), and several social benefits, including disability payments (64%) and social security (59%). Few veterans used food assistance programs, such as SNAP (25%), free meal/soup kitchens (12%), and free/reduced-price meals for their children at school or in childcare (6%). The sum of self-reported programs used ranged from 0 to 6, with half of the sample using no more than two types of resources. The majority of the sample was food secure (60%).

Grit and help-seeking scores are shown in Table 3. The mean grit score of the sample was 3.5 out of 5 and the mean help-seeking score was 3.5 out of 7.

**Figure 1:**
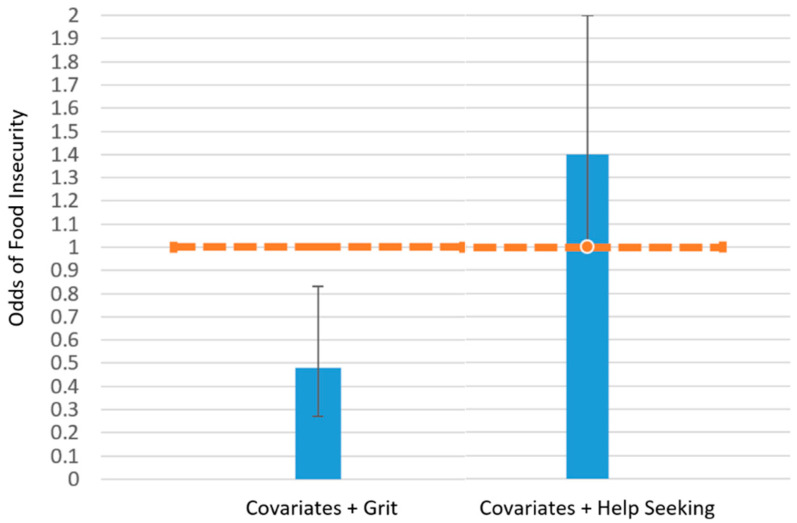
Grit, but not help-seeking, was inversely associated with the likelihood of being food insecure among rural Illinois veterans ≥18 years recruited from five food pantries from March 2021 to November 2021. (Logistic regression models with imputed data were used and adjusted by age and sex; *n* = 162 for food security, *n* = 170 for grit, and *n* = 164 for help-seeking were included for analysis). Orange dashed line indicates an odds ratio of 1 as reference; orange box highlights significant results.

Figure 1 shows the association between the odds of food insecurity with grit and help-seeking, respectively. The odds ratio of grit in the adjusted regression model was 0.47, indicating that one point higher in grit score was associated with 53% lower odds of being food insecure. There was no significant association found between the help-seeking score and the odds of being food insecure.

Lastly, the regression model results between resource use with grit and help-seeking, respectively, are presented in Table 4. Neither grit nor help-seeking were associated with resource use. Similarly, modeling with the original data without imputation did not generate significant results.

## 4. Discussion

The sample of veterans in this study were primarily white, male, and over 65 years old. Around 40% were food insecure and more than half of the participants used two or fewer assistance programs or resources, indicating the success of the RRV program in reaching rural veterans with high needs. Higher grit was associated with less risk of being food insecure but not resource use, while help-seeking was not associated with either food security or resource use. Compared to the general veteran population [43], the current sample was similar in race and sex but different in other socio-demographic characteristics. The current sample had a higher percentage of veterans over 65 years, with lower education attained (college degree or above), higher service-related disabilities, and lower income compared to the general veteran population [43].

The 40% food insecurity rates found here were higher than the results of veteran groups documented in previous studies, which was expected considering the nature of RRV to engage with rural veterans of low resources. A previous study on veterans in wars in Iraq and Afghanistan [7] and another study designed for observing determining factors on the clinical outcomes of HIV infection among veterans [8] both found a food insecurity rate of around 25% among the veteran population assessed. Another study on working-age (18–59 years) veterans with children found that around 17% of veterans were food insecure [44]. These authors also reported that even though the odds of food insecurity were not higher among veterans, they were more likely to have more severe food insecurity associated with hunger compared to nonveterans [44]. The results in the present study similarly show high rates of very low food security, where the amount of food is reduced due to a lack of resources for food, at 54% among those with food insecurity in the sample. The high food insecurity rate and percentage with very low food security in the present study were likely a result of the recruitment at food pantries, where those with limited access to resources for food are more likely to visit. Additionally, this project featured a sample of rural veterans, where resources may be less available and also where only one previous project has specifically focused and reported on food security. The prior RRV pilot project reported a higher food insecurity rate (70%) among rural veterans in Indiana and Kentucky compared with the current sample [34]. Both studies highlighted the high risk of being food insecure among rural veterans using food pantries. The present study results were very similar to the 49% food insecurity reported among a sample of veterans from Veterans Administration Clinics [45]. The high prevalence estimates of food insecurity also documented the contribution of other risk factors to food insecurity, including poor health conditions (i.e., diabetes and prediabetes) [45] and sociodemographic characteristics such as being women and/or racial or ethnic minorities [46].

Such few previous U.S. studies have importantly documented this under-studied topic of veteran food insecurity, a topic with even less prior investigation in countries outside of the U.S. Only one study from an international context, the United Kingdom (U.K.), evaluated food insecurity among veterans (median age group was over 55 years old) and found that around 10% were living in food-insecure households, while those with low income and disabilities were especially at risk for food insecurity [47], consistent with findings among the general population of U.S. veterans [46]. Such documentation provided evidence of the unmet needs of veterans outside of the U.S. and highlighted the need for further investigation of veteran food security and needs globally.

Despite the high rates of food insecurity, resource use among the sample in the present study was low, which was also consistent with the previous RRV pilot project [34]. Although a majority of the veterans reported using veteran-specific benefits and services, very few reported using food-related assistance programs such as SNAP, free meal/soup kitchens, and free/reduced-price meals at school/childcare. Specifically, the prevalence of using SNAP reported in the present sample was low (25%). This was consistent with a previous study on low-income veterans, where the authors estimated a 27% SNAP participation rate [48], but lower than what was reported in the previous RRV (33%) [34], while another report found around 22% SNAP participation among low-income veterans [49]. The SNAP income threshold for ages 60 and over is $2873 per month, which approximates $34,476 per year [50]. The characteristics of this sample where 65% are over 65 years show that 60% have an annual income below $30,000. Thus, a majority of the sample may likely be eligible for SNAP, and the reported low SNAP participation rate represents a potential gap between SNAP eligibility and participation among the sample, which may also be present among other rural veterans. Food pantries could serve as an important link to connect rural veterans who visit the pantry to SNAP, with the aim of encouraging SNAP enrollment and closing the potential eligibility-to-participation gap among this at-risk group.

Very limited research on veterans in other countries has supported the importance of connecting resources with veterans. A study on Chinese elderly veterans showed that poor health and inadequate income were the main reasons provided regarding life dissatisfaction and that existing social security and benefits were insufficient and required improvements [51]. On the other hand, veterans in the U.K. had higher food security when receiving disability benefits compared to those without benefits [47]. This evidence indicated a potentially similar shared need among veterans globally and the importance of being connected with the resources they qualify for. Veteran outreach strategies to link veterans with resources, such as the RRV program, could be adapted and utilized in designing and delivering interventions and assistance programs for veterans in other countries, to improve veteran use of resources and quality of life.

The current investigation was carried out as a part of the RRV pilot intervention, a longitudinal project designed to test intervention methodologies to improve the connections and utilization of resources and food security status for rural low-resource veterans and family members through outreach events such as resource fairs and other interventions. The low participation in assistance programs such as SNAP reported in the current manuscript highlights the high need for connection to resources among this rural veteran population and justifies the necessity of RRV programs. The results also provide insight and guidance for future RRV interventions to focus on connecting veterans with these resources using food pantries as delivery sites. The resource use status assessed in the current project provides a baseline for resource use, which is aimed to be improved with RRV and would be valuable for RRV evaluation after the intervention is delivered.

Grit scores were inversely associated with the odds of food insecurity. This finding was consistent with previous studies [25,29] among non-veteran populations. Further explanation of this association is needed, as causation cannot be inferred from the cross-sectional design. It is not clear if high grit has led to a lower risk of food insecurity or if food security occurred before grit was developed, and perhaps experiences of food insecurity may lessen grit. Additionally, it is unknown if grit alone was impactful in this association or if grit is an indication of other potential beneficial qualities. Grit might be a marker for other personal traits or behaviors that are advantageous against adverse situations, such as food-obtaining behaviors, participation in assistance programs, the seeking and use of available resources, and budgeting. Individuals with these traits may be less likely to experience situations of need. Future research that explores these behaviors is needed to better understand the meaning of this association.

Previous studies among the general population of U.S. veterans have also found that food insecurity was associated with several psychological factors, including depression, suicidal thoughts, anxiety, and stigma related to COVID-19 infection [52,53]. Other studies reported associations between food security with little sleep, medical and trauma-related comorbidities, and housing instability among veterans [46,54]. Not unique to the U.S., veterans worldwide tend to have chronic physical and mental conditions, as well as low income [47,51,55,56,57,58,59]. Veterans were more likely to suffer from chronic illnesses such as arthritis, depression, anxiety, post-traumatic stress disorder, and low general well-being in the U.S., Canada, Australia, the U.K., and China [47,51,55,56,57,58,59]. Health and well-being also tend to worsen over time since leaving military service [59]. Considering the interconnection of physical, mental, and social health [60] and the shared conditions among veterans internationally, more research is imperative to fully understand the psychological, health, and social factors associated with food insecurity among veterans to better address these needs among this at-risk population. The results reported here provide insights to inform tailored interventions and support for veterans.

Interestingly, help-seeking was not associated with either food security or resource use. As this was a self-reported measurement, veterans may feel stigma in reporting and/or performing help-seeking behaviors, which was one of the reported barriers to seeking help among veteran groups [15]. Additionally, the raw responses from the help-seeking questionnaire contained a large number of missing data. For example, field staff reported that many veterans left certain questions empty, such as the potential help sources, because individuals (i.e., parents) had passed away. Considering the age distribution of the current sample, the “parent” option might not be a suitable source to include in assessing the help-seeking behaviors of the sample, which contributed to some of the missing responses to this question. As mentioned, the authors imputed responses to retain the use of the data despite the missing entries for help-seeking, along with grit and food security. Yet, the conservative treatment of the imputation may have muted potential links of help-seeking with food security or resource use. Future studies should explore creative strategies and instruments that engage rural veterans and consider shorter questionnaires to improve completion rates.

There were limitations to the current study. As mentioned previously, there were a large amount of missing data for the outcome measurements. Although statistical power was retained with imputation, the conservative procedures could have biased the association to null. Future studies should focus on improving the clarity and conciseness of the survey questions, making sure field staff are available for answering questions, quality checking to ensure the completion of surveys, and reducing the amount of missing data. The current investigation also did not capture purchasing and budgeting behaviors or attitudes and barriers to using assistance programs in the sample, which may be additional factors involved in the relationships between grit, help-seeking, and food security that may be important to adjust in the regression models and/or further explain the association between grit and food insecurity found in the current analysis. Future research on this topic should incorporate these variables. The sample was also recruited through food pantries, which may mean that the results are not generalizable to the broader rural veteran population. However, there is a lack of research on the food security of the rural veteran population and their resource-use status. Previous studies on help-seeking behaviors have been mostly focused on academic performance, mental health, or physical health conditions [15,18,20,22,23,61,62] and have not assessed the link to food security or resource use. Therefore, despite limitations, the results represent a first look at help-seeking in rural veterans. The current project could serve as a reference for future studies focusing on rural veteran populations and provide insights for better study designs and recruitment strategies.

The current results are the first to describe the psychological traits of grit and help-seeking with food insecurity and resource use among a sample of rural veterans of high need. These findings may inform future policies and proposals. For example, the determination of the association specifically between grit and food insecurity from the results supports the justification of the funding of future interventions or assistance programs to foster grit, for food security improvement and connecting veterans with health resources that they may be eligible for. Policymakers might also consider supporting intervention programs that include one-on-one coaching or group sessions to develop and strengthen a growth mindset and skills to help bolster persistence to improve grit [19,27,28]. The results also reveal the high needs among the rural veteran groups using food pantries. Resource fairs, such as RRV, where program representatives provide program information and offer assistance in the enrollment process are shown in these results to successfully attract the intended audience. Similar future events might be helpful in connecting veterans with resources they are eligible for but not currently using, promoting help-seeking behavior, and ultimately improving resource use. Such interventions are advantageous, as education may sustain impact and provide benefits in the long-term future after the intervention is over [63]. Enhancing psychological traits such as grit may improve the mental health of veterans and family members, improving their quality of life independently from food security and potentially supporting improvements of both physical and mental health that could ease the healthcare burden on both the household and healthcare system. Therefore, the findings from the current project provide novel insights into rural veteran groups using food pantries and have important applications for future research, intervention development, and policies. In summary, salient applications are: 1. interventions that foster grit may also foster food security among rural veterans; 2. targeted outreach to rural veterans at food pantries can attract a group with high needs and low access to food and other resources; 3. determining and addressing the gap between eligibility and participation in assistance programs may inform future interventions to improve food security among rural veterans.

## 5. Conclusions

Grit was inversely associated with the odds of food insecurity among a cross-sectional sample of rural veterans. The results provided evidence to inform the content of future educational interventions to improve food insecurity and address health disparities among rural veterans by addressing grit. The enhancement of psychological traits such as grit might improve quality of life independently from food security and potentially benefit other aspects of well-being.

## Figures and Tables

**Table 1:** Socio-demographics among rural southern Illinois veterans ≥18 years recruited from five food pantries from March 2021 to November 2021.

Variables		N	% ^1^
Age			
	18–44 years	16	9.0
	45–64 years	46	26.0
	≥65 years	115	65.0
Sex			
	Male	156	88.6
	Female	20	11.4
Race			
	White	162	92.0
	African American	7	4.0
	American Indian or Alaska Native	4	2.3
	Other	3	1.7
Education level			
	High school, equivalent or less	69	40.8
	Some post-high-school education but below college	85	50.3
	College and above	15	8.9
Employment status			
	Employed	26	14.8
	Unemployed	9	5.1
	Not in labor force	141	80.1
Marital status			
	Married/living with partner	100	56.5
	Widowed	20	11.3
	Divorced/separated	49	27.7
	Never married	8	4.5
Household type			
	With children <18 years	27	15.7
	Without children <18 years	145	84.3
Household size			
	1 adult	50	29.1
	2 adults	103	59.9
	≥3 adults	19	11.1
Household income in the last 12 month			
	≤$15,000	21	27.6
	$15,000–$30,000	25	32.9
	>$30,000	30	39.5
Military status			
	Veteran	156	91.8
	Non-active	13	7.7
	Active	1	<1
Branch of military			
	Air Force	12	6.9
	Army	117	67.2
	Marine Corps	10	5.8
	Navy	32	18.4
	Multiple branches	3	1.7
Guard/Reserve Service			
	Yes	52	30.2
	No	120	69.8
Years served	mean (SD)	5.6 (5.8)	
Service-related Veterans Affairs-recognized disability			
	Yes	57	33.0
	No	116	67.0
Service-related non-Veterans Affairs-recognized disability			
	Yes	52	30.0
	No	121	69.9

^1^ Totals may not add to total n and percentages may not add to 100 due to missing values and rounding.

**Table 2:** Resource use and food security status among rural Illinois veterans ≥18 years recruited from five food pantries from March 2021 to November 2021.

	Status	N	%
Veterans Affairs healthcare			
	Yes	109	77.3
	No	32	22.7
Veteran Affairs disability benefits			
	Yes	56	59.6
	No	38	40.4
Veteran pension			
	Yes	35	21.1
	No	131	78.9
Disability payments			
	Yes	57	36.5
	No	99	63.5
Employer pension/retirement fund			
	Yes	37	22.6
	No	127	77.4
Social security			
	Yes	100	58.5
	No	71	41.5
TANF ^1^			
	Yes	2	1.2
	No	160	98.8
Employment compensation			
	Yes	6	3.7
	No	156	96.3
General assistance from the township trustee			
	Yes	1	0.6
	No	161	99.4
SNAP ^2^			
	Yes	42	25.2
	No	125	74.9
Free meals, soup kitchens			
	Yes	19	11.7
	No	143	88.3
Free/reduced-price meals at school/childcare			
	Yes	10	6.2
	No	152	94.8
Sum of all programs reported			
	0	21	11.9
	1	23	13.0
	2	40	22.6
	3	41	23.2
	4	25	14.1
	5	14	7.9
	6	13	7.3
Food security ^3^			
	Food secure	97	59.9
	Food insecure	65	40.1
	Low food security ^4^	30	46 ^5^
	Very low food security ^4^	35	54 ^5^

^1^ TANF, Temporary Assistance for Needy Families. ^2^ SNAP, the Supplemental Nutrition Assistance Program. ^3^ Food security results including imputed data are presented. ^4^ Category “food insecure” was further broken into “low food security” and “very low food security”. ^5^ Percentage was calculated based on the number of participants categorized as “food insecure”.

**Table 3:** Grit and help-seeking scores among rural Illinois veterans ≥18 years recruited from five food pantries from March 2021 to November 2021 ^1^.

Variables	Scores (Mean ± SD)
Grit (1–5 score)	3.50 ± 0.67
Help-seeking (1–7 score)	3.48 ± 1.00

^1^ Scores were calculated based on available data with imputation. Total sample size including imputed data: *n* = 170 for grit; *n* = 164 for help-seeking. The imputation was completed for those with missing values for less than 20% of the responses and the missing items were imputed as the mean of the available responses, for calculating the sum scores.

**Table 4:** Grit and help-seeking were not associated with resource use among rural Illinois veterans ≥18 years recruited from five food pantries from March 2021 to November 2021 ^1^.

Variable	Estimates ± SE	
	Covariates + Grit	Covariates + Help-Seeking
Resource use (0–6 programs)	−0.023 ± 0.07	0.014 ± 0.05

^1^ Poisson regression models with imputed data were used. Covariates adjusted included age and sex; *n* = 162 for food security, *n* = 170 for grit, and *n* = 164 for help-seeking were included for analysis.

## Data Availability

The data presented may be discussed for potential use with the corresponding author.
